# Enhancement of Neoangiogenesis and Follicle Survival by Sphingosine-1-Phosphate in Human Ovarian Tissue Xenotransplants

**DOI:** 10.1371/journal.pone.0019475

**Published:** 2011-04-29

**Authors:** Reza Soleimani, Elke Heytens, Kutluk Oktay

**Affiliations:** Laboratory of Molecular Reproduction, Institute for Fertility Preservation, Departments of Obstetrics and Gynecology and Cell Biology and Anatomy, New York Medical College, Valhalla, New York, United States of America; National Cancer Institute, United States of America

## Abstract

Ovarian transplantation is one of the key approaches to restoring fertility in women who became menopausal as a result of cancer treatments. A major limitation of human ovarian transplants is massive follicular loss during revascularization. Here we investigated whether sphingosine-1-phosphate or its receptor agonists could enhance neoangiogenesis and follicle survival in ovarian transplants in a xenograft model. Human ovarian tissue xenografts in severe-combined-immunodeficient mice were treated with sphingosine-1-phosphate, its analogs, or vehicle for 1–10 days. We found that sphingosine-1-phosphate treatment increased vascular density in ovarian transplants significantly whereas FTY720 and SEW2871 had the opposite effect. In addition, sphingosine-1-phosphate accelerated the angiogenic process compared to vehicle-treated controls. Furthermore, sphingosine-1-phosphate treatment was associated with a significant proliferation of ovarian stromal cell as well as reduced necrosis and tissue hypoxia compared to the vehicle-treated controls. This resulted in a significantly lower percentage of apoptotic follicles in sphingosine-1-phosphate-treated transplants. We conclude that while sphingosine-1-phosphate promotes neoangiogenesis in ovarian transplants and reduces ischemic reperfusion injury, sphingosine-1-phosphate receptor agonists appear to functionally antagonize this process. Sphingosine-1-phosphate holds great promise to clinically enhance the survival and longevity of human autologous ovarian transplants.

## Introduction

Chemotherapy-induced infertility and ovarian failure are significant quality of life issues in women. Ovarian cryopreservation prior to chemotherapy followed by auto-transplantation has been performed to preserve and restore fertility in cancer survivors [1]–[8]. Because ovarian grafts are transplanted against a vascular bed on the pelvic sidewall [1], subcutaneously [2], [3], or on the remaining menopausal ovary [4], [5] their survival is acutely dependent on the neovascularization process, akin to skin grafting. The initial ischemic phase, which occurs until the revascularization process is completed is associated with massive primordial follicle loss [9] and limits the longevity and success of ovarian transplants (OT) [10], [11]. Therefore, there is a great clinical need to develop pharmacological strategies to enhance neoangiogenesis after ovarian transplantation and to improve the clinical utility of this procedure.

Sphingosine-1-phosphate (S1P) is a sphingolipid metabolite which was originally shown to inhibit germ cell apoptosis induced by radiation and chemotherapy in mice [12]. Recent work also implicated S1P as a modulator of endothelial cell migration [13], [14], and angiogenesis in non-human non-reproductive tissues [15]. It has been shown that S1P can provoke the formation of cell-cell aggregates in differentiated endothelial cells [16] that act as a predominant angiogenic stimulus by prompting vascular endothelial cell growth, migration, and tube formation [17], [18].

To date, five S1P receptors have been identified and cloned. These S1P receptors are originally known as the endothelial differentiation gene-1 (EDG-1) family of proteins [19] and include S1P1 (EDG-1), S1P2 (EDG-5), S1P3 (EDG-3), S1P4 (EDG-6) and S1P5 (EDG-8). The diverse effects of S1P are tissue specific which is explained by some to be the result of variations in heterotrimeric G proteins downstream of S1P receptors [20] or by its actions as an intracellular messenger [21]. Previous studies indicated that the angiogenic effect of S1P is mostly receptor driven but others suggested an intracellular messenger mechanism [17], [22], [23]. Receptor mediated angiogenic effect of S1P is mainly through S1P1 [16] and to some extent with S1P2-5 [24] receptors. Signal cross-talk between S1P1 and angiogenesis related growth factor receptors, such as the platelet-derived growth factor [25] and VEGF [6] has also been demonstrated.

Recently, several synthetic S1P analogs have become commercially available. One such agent is SEW2781, which is a selective S1P1 receptor agonist. FTY720 is another S1P agonist which is currently used in clinical trials as an immunosuppressant for renal transplantation [26], [27]. By virtue of its immunosuppressive effects, FTY720 has also been demonstrated to improve allograft survival in various solid organ allo-transplant models such as liver [28] and intestine [29]. FTY720 is phosphorylated *in vivo* by sphingosine kinase and the phosphorylated form is a potent agonist of S1P1, 3, 4 and 5 receptors [30].

Given the recent evidence that S1P may play a positive role in angiogenesis in non-reproductive cell lines and tissue [13], [15], [23], [31], [32], [33], we hypothesized that S1P and its agonists could improve neoangiogenesis, and as a result, primordial follicle survival during human ovarian cortical tissue transplantation. To study the impact of S1P on OTs *in vivo*, we used a xenograft model previously developed by us [34], [35], [36].

## Results

### Comparison of angiogenic effects of S1P, FTY720 and SEW2871

**Figure 1:**
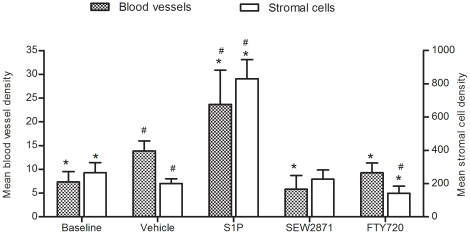
The effect of S1P, SEW2871 and FTY720 on ovarian transplant revascularization and stromal cell population. S1P induces ovarian angiogenesis (Left axis) and increases stromal cell population (Right axis) while its analogs, SEW2871 and FTY720 have the opposite effect, 10 days after grafting of ovarian transplantation. * Significantly different from control. # Significantly different from baseline ovarian tissue.

**Figure 2:**
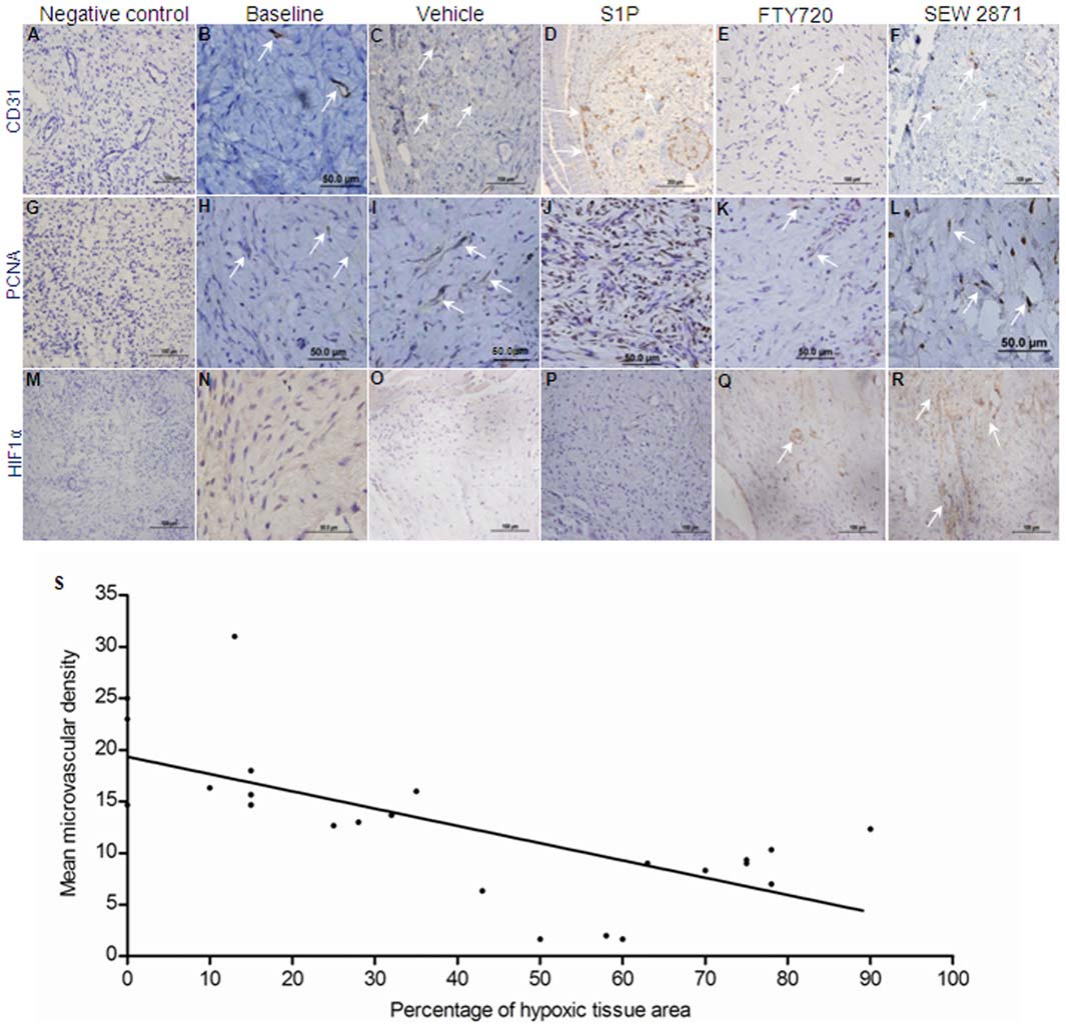
Impact of S1P and its analogues on neo-angiogenesis, stromal cell proliferation and tissue hypoxia. (**A–F**) Evaluation of angogenic effect of S1P and its analogs in ovarian transplants by anti-CD31 IHC. S1P induces angiogenesis of ovarian transplants (**D**) while FTY720 (**E**) or SEW2871 (**F**) treatment reduces it. Arrows show newly formed blood vessels positively stained for anti-CD31. (**G–L**) Evaluation of cell proliferation by anti-PCNA expression. S1P induces (**J**) where as FTY720 (**K**) reduces stromal cell proliferation. SEW2871 (**L**) treatment does not affect stromal cell proliferation compared to vehicle-treated control grafts. Arrows show proliferating stromal cell positively stained for PCNA. (**M–R**) Evaluation of ovarian tissue hypoxia by HIF1α.While S1P (**P**) treatment reduces tissue hypoxia compared to vehicle-treatment. FTY720 (**Q**) and SEW2871 (**R**) treatment is associated with massive tissue hypoxia 10 days post-grafting. (**Q**) Arrow shows hypoxic ovarian follicles in a FTY720-treated graft. (**S**) There is an inverse correlation between vascular density and hypoxic ovarian tissue surface area in the grafts, as determined by HIF1α staining. This indicates that enhanced vascular density reduces hypoxia in ovarian transplants.

To determine the effect of S1P and its analogs on neoangiogenesis we used anti-CD31 (PECAM-1) antibody as a surface marker of new blood vessels [37]. Angiogenesis was more prominent in S1P-treated OTs in comparison with the vehicle-treated controls (23.7±7.2 vs. 13.9±2.1; p = 0.001) (Figures 1 and 2A–D). In contrast, FTY720 and SEW2871 treatment was associated with reduced vascular density compared to vehicle-treated controls (9.3±2.1 and 5.8±2.9 respectively, vs. 13.9±2.1; p<0.001) (Figures 1 and 2E,F).

S1P treatment also affected SC population. While there was a significant decrease in SC density in vehicle-treated controls compared to the baseline (200.9±28.5 vs. 265.0±61.5; p<0.001) (Figure 1), S1P-treated grafts showed significantly higher SC density compared to the baseline, the vehicle-treated control, and any other experimental group (830.4±115.1; p<0.001) (Figure 2G–L). There was no difference between the vehicle-treated control and the SEW2871-treated tissues in the SC density (200.9±28.5 vs. 226.3±55.8; p>0.05). FTY720 treatment resulted in the lowest SC density of all experimental groups (142±43; p<0.001) (Figure 2K and Figure S1) while there was massive SC proliferation in S1P-treated ovarian transplants as demonstrated by PCNA staining. In contrast, a higher fraction of OTs contained non-uniform acellular/hypocellular areas in the untransplanted controls compared to S1P-treated OTs (16.7% vs. 0%, p = 0.007). FTY720 and SEW2871 treatment was associated with a larger fraction of grafts with >25% acellular areas (less than 30 cells/grid area under high power field) compared to S1P (100% and 83.3% vs. 0% respectively; p<0.05).

Because xenografted ovarian tissues are small and O_2_ diffusion from peripheral tissues is conceivable, one can question whether increased vascular density would actually improve tissue perfusion. By HIF1alpha expression in the grafted tissues as an endogenous hypoxia marker [38], S1P-treated OTs demonstrated significantly reduced tissue hypoxia compared to vehicle-treated controls on day 10 (6.9±6.6% vs. 25±8.5% hypoxic area; p<0.05) (Figure 2M–P) whereas FTY720 and SEW2871 treatment was associated with massively increased tissue hypoxia compared to vehicle-treated controls (75.7±9 and 57.2±12 vs. 25±8.5 respectively; p<0.05) (Figure 2Q,R). Overall, vascular density and the percentage of hypoxic tissue area showed a strong negative correlation (Spearman's rank correlation coefficient: −0.764, p<0.001) indicating that improved revascularization results in the reduction of tissue hypoxia (Figure S2).

To demonstrate that S1P is enhancing neoangiogenesis by inducing proliferation of the existing human blood vessels, we studied alphaSMA expression. Anti-alphaSMA IHC demonstrated a significantly lower density of mature blood vessels in S1P- compared to vehicle-treated control, FTY720- and SEW2781-treated OTs (2.0±1.2 vs. 5.1±0.8, 5.6±1.3 and 4.1±1.8 respectively; p<0.05) (Figure S2) indicating that S1P induced proliferation and formation of new blood vessels as opposed to enhancing the survival of pre-existing ones. However, adjacent sections of CD31 positive vessels were also stained for alphaSMA in most of the S1P-treated grafts, indicating that S1P can induce neoangiogenesis from pre-existing mature vessels (Figure S2E,F).

### Impact of S1P and its analogs on follicle survival

We then investigated whether enhanced neoangiogenesis improved survival of primordial follicles (n of donors = 3; n of grafts in each group S1P = 8, FTY720 or SEW2871 = 12). There was a significant decrease in the percentage of apoptotic follicles in S1P-treated xenografts compared to the vehicle-treated control- and SEW2871- or FTY720-treated groups (6.8±10.7 vs. 11.8±5.7, 15.9±4.2 and 17.9±4.0 respectively, p≤0.001). Compared to vehicle-treated controls, SEW2781 or FTY720 did not have an impact on the percentage of apoptotic follicles in OTs, indicating that S1P but not its analogs improves follicle survival. Overall, vascular density and the percentage of apoptotic follicles showed a negative correlation (Spearman's rank correlation coefficient: −0.257, p<0.01) in all groups confirming the dependency of follicle survival on ovarian angiogenesis.

### Does S1P accelerate neoangiogenesis?

**Figure 3:**
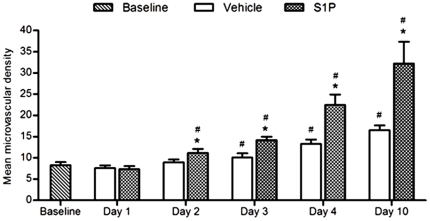
Impact of S1P on early angiogenesis after ovarian transplantation. S1P-treated ovarian transplants show significantly higher density of microvasculature by as early as two days after transplantation. * Significantly different from matching vehicle-treated control. # Significantly different from baseline.

Having shown that S1P but not its analogs improve ovarian neoangiogenesis, we then investigated whether S1P treatment accelerated this process (n of donors = 1; n of grafts in each group  = 12). While we did not find a significant difference in the density of CD31-positive blood vessels between the vehicle-treated control grafts and the baseline before day-3 after xenotransplantation, this difference was detectable starting two days after xenotransplantation in the S1P-treated grafts (p = 0.001) (Figure 3). Even though grafts in both S1P- and vehicle-treated control groups demonstrated a significant rise in the density of CD31-positive blood vessels by day-10 compared to baseline, the vascular density was doubled in the S1P- compared to the vehicle-treated group (32.2±5.2 vs. 16.5±1.1 respectively; p<0.001). The comparison of follicle survival was not possible in this experiment due to the low baseline follicle density.

By HIF1alpha staining, 100% of the surface area of OTs in S1P- and vehicle-treated control groups demonstrated hypoxia during the first four days after grafting but the intensity of staining was lower in the S1P-treated OTs. The presence of hypoxia is expected as these microvessels are known to become functional as late as by day-4 [36], [39], [40]. However, hypoxia was reduced by day-10 in the S1P-treated OTs compared to vehicle-treated controls (5.6±6.3 vs. 20.8±6.8 respectively; p = 0.001).

### Determining the contribution of the recipient site to transplant revascularization using an acellularized extracellular scaffold

Next, to determine whether S1P induces neoangiogenesis from the recipient site, OT, or both, we substituted OTs with an acellular human extracellular matrix scaffold (Alloderm). A significantly higher density of murine blood vessels was detected in S1P-treated versus vehicle-treated control alloderm grafts on day-10 (6.4±0.5 vs. 4.2±0.7 respectively; p<0.005). There was significant cell proliferation and migration into alloderm in both vehicle-treated control and S1P-treated groups, but this was more prominent in S1P-treated group (139±7.4 vs. 62.3±3.8 respectively; p<0.005) (Figure S3). These findings indicate that, S1P can induce neoangiogenesis from the recipient tissue even in the absence of ovarian grafts and S1P targets both the implantation site and ovarian tissue to enhance graft survival.

### Is S1P-induced neoangiogenesis and improved follicle survival affected by ovarian tissue cryopreservation and thawing process?

In the majority of fertility preservation cases, ovarian tissue is cryopreserved before chemotherapy, and thawed later for transplantation. Therefore, next we repeated the S1P- experiments using frozen-thawed ovarian tissue to determine whether S1P had the same beneficial effect on ovarian neoangiogenesis in frozen-thawed tissue.

First, we did not find any difference in the density of CD31-positive vessels between the fresh-fixed and cryopreserved-thawed and fixed baseline ovarian tissues (5.5±0.6 vs. 5.7±0.6; p>0.05), indicating that cryopreservation process itself did not morphologically affect pre-existing vascular structures. We then found that S1P resulted in a significantly larger increase in vascular density compared to vehicle-treated controls (28.5±3.7 vs. 12.3±0.8, p = 0.001) after 10 days of xenografting when frozen thawed ovarian tissue were used. This improvement was similar to that seen with fresh ovarian transplants (Figure 1), hence proving that S1P has the same pro-angiogenic effect both in fresh and frozen-thawed grafts.

### What is the impact of S1P on follicle survival in frozen-thawed ovarian transplants?

**Figure 4:**
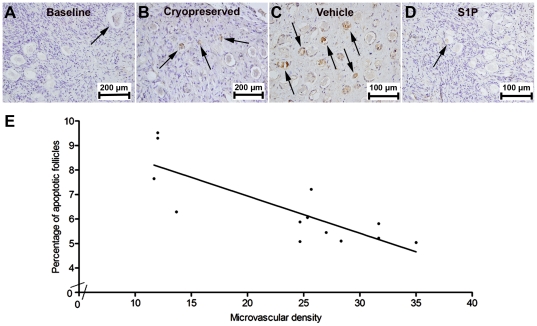
Impact of S1P on follicle survival in frozen-thawed ovarian transplants. Frozen-thawed ovarian tissue was transplanted to SCID mice and percentage of apoptotic follicles were compared between S1P-treated and vehicle-treated control grafts. (**A–D**) Histomorphological evaluation of apoptosis induced by cryopreservation and/or transplantation. The percentage of apoptotic follicles was significantly lower in fresh baseline ovarian tissue (**A**) compared to cryopreserved-untransplanted ovarian tissue (**B**) indicating that cryopreservation process itself induces apoptotic ovarian follicle death. S1P treatment results in significantly decrease in the density of apoptotic follicles (**D**) compared to the vehicle-treated group (**C**). Arrows indicate apoptotic ovarian follicles. (**E**) Inverse correlation of apoptotic follicle death with vascular density indicating that improved vascularization by S1P enhances follicle survival.

To demonstrate the impact of S1P on follicle survival in an experimental setting similar to clinical trials [1], [3], [11], ovarian tissue from a two-year-old (n = 8 grafts in each group) was slow-frozen, thawed and grafted as above described. AC3 IHC was used as a marker of follicular apoptosis. The percentage of apoptotic follicles was significantly higher in cryopreserved OTs compared to the untransplanted tissue at baseline (14.6±3.4 vs. 3.3±0.6, p = 0.004), indicating that cryopreservation process itself induces apoptotic follicle death. S1P treatment significantly lowered the percentage of apoptotic follicles with frozen-thawed OTs compared to xenografted vehicle-treated controls (5.7±0.7 vs. 8.2±1.5; p = 0.006) (Figure 4A–D).

### Does S1P improve follicle survival in OTs independent of its antiapoptotic effects?

Previous studies have shown that S1P can rescue ovarian primordial follicles from apoptotic death [12]. To verify that improved neovascularization but not direct anti-apoptotic effects of S1P resulted in reduced percentage of apoptotic follicles in OTs, we correlated the vascular density with percentage of apoptotic follicles. A significant inverse-correlation was observed between the density of blood vessels and percentage of apoptotic follicles in the xenografts (Spearman's rank correlation coefficient:−0.77; p = 0.002) (Figure 4E), indicating that S1P-induced neoangiogenesis directly contributed to post-ovarian transplant follicle survival. Further, because tissue hypoxia showed a strong negative correlation with vascular density (Figure S2), we concluded that S1P treatment enhances follicle survival in OTs by reducing tissue hypoxia by way of increased vascular density.

## Discussion

Despite recent success in the development of ovarian tissue cryopreservation and transplantation techniques, massive loss of primordial follicles during the initial days after the ovarian transplantation limits the success of the procedure. Ischemic reperfusion injury induced by oxygen-derived free radicals and lipid peroxidation leading to primordial follicle loss have been cited as the most significant drawback of human ovarian tissue transplantation [41]. Others have suggested that prevention of oxidative stress during transplantation will lead to prevention of ischemic reperfusion injury and increase ovarian follicle survival consequently [10].

Given the recent preliminary evidence that S1P may enhance neoangiogenesis, we studied the effect of S1P on early angiogenesis after human ovarian transplantation that is crucial for the graft survival. Our data showed that S1P not only enhanced the mean density of newly formed blood vessels in OTs, but it also accelerated neoangiogenesis compared to vehicle-treated controls. This acceleration of neoangiogenesis by S1P is expected to be of high clinical significance because the longer the initial ischemia the higher will be the follicle losses from OTs. Consistent with our hypothesis, we found that the enhancement of ovarian angiogenesis was associated with reduced tissue hypoxia and a lower percentage of apoptotic follicles.

After 10 days of xenografting, the higher density of CD31-positive blood vessels and the reduced number of mature blood vessels stained by alphaSMA in S1P-treated grafts indicated enhanced proliferation of new blood vessels. There was a smaller but significant increase in vascular density in untreated xenografts compared to baseline on day-10, which can be explained by the wound healing process in the grafts. Similar results were obtained after OT with cryopreserved human tissue, indicating that our cryopreservation protocol did not affect the neoangiogenesis in OTs.

Because S1P has a well-known direct anti-apoptotic effect on oocytes [12], one could question whether the benefit of S1P is via enhanced angiogenesis, improved oocyte survival, or both. First, we showed in earlier studies that ovarian follicle growth in human ovarian xenografts correlates with vascular density [36]. Second, in the current work, we analyzed the correlation between vascular density, apoptotic follicle death, and tissue hypoxia. We found that the vascular density was inversely correlated with the percentage of hypoxic tissue and apoptotic follicles in OTs. Therefore, while a contributory direct anti-apoptotic effect on follicles cannot be ruled out, our data indicates that S1P protects follicles in OTs by enhancing the revascularization process.

To study the impact of S1P on regeneration of vascular structures from the recipient site in isolation, we substituted ovarian transplants with alloderm, an acellular extracellular scaffold. Because alloderm is immunologically inert and contains no cellular or vascular structures [42], any cells or blood vessels which were detected after grafting would have to originate from the recipient. We found that S1P significantly stimulated neoangiogenesis and SC proliferation from the murine dorsal muscles even in the absence of ovarian tissue. The latter effect may indicate that S1P's action is both on the recipient site and the ovarian graft and thus S1P may also be beneficial in improving ovarian transplantation to heterotopic locations such as subcutaneous [3]. This finding also indicates that S1P treatment may also be useful in improving neoangiogenesis in other non-reproductive tissue grafting and organ transplant settings.

Surprisingly, and in contrast to S1P, S1P receptor agonists impeded neovascularization and induced tissue hypoxia. Reduction of angiogenesis by SEW2871, a specific agonist of S1P1, suggests that the pro-angiogenic effect of S1P1 in human ovarian tissue maybe via a non-receptor mechanism. It is possible that similar to its action in bovine aortic endothelial [17] and muscular smooth muscle cells [22], [23], S1P1 acts primarily as an intracellular messenger to promote neoangiogenesis in OTs.

Consistent with the previous data showing that FTY720 has anti-angiogenetic effects in a mouse melanoma model [43] we also found that FTY720 inhibited angiogenesis and induced hypoxia in OTs. Currently, ovarian transplantation is performed with autologous or genetically identical tissue but it has been hypothesized that this procedure can be performed among genetically non-identical individuals when safer immune-suppression agents such as FTY20 have become available. Even though FTY720 is proven to be effective in preventing organ rejection after liver [28] and kidney transplantation [44], [45], [46], it may not be suitable in OT transplantation due to its antiangiogenic effect. Interestingly, FTY720's immunosuppressive effect, which is due to its inhibition of T-lymphocyte infiltration, also appears to be receptor-independent [47]–[50].

Ovarian SC [51] and ovarian extracellular matrix have been reported to have an important role in normal ovarian function, follicle growth, and survival [52], [53]. We observed a significant reduction of the SC population in grafted OTs compared to the baseline. S1P treatment enhanced SC survival and proliferation, whereas FTY720 had the opposite effect. This indicates that in addition to enhancing angiogenesis, S1P treatment may offer a supplementary mechanism of support for OT survival.

It can be questioned whether different dosing of S1P agonists could have changed the outcome. We are doubtful that the dose we used was ineffective as both agonists reduced the vascular density compared to vehicle controls confirming a real biological effect. If the local concentration were ineffective, we would have seen no biological effect by the agonists. Further pointing towards the specificity and the existence of the action by the agonists, FTY720 and SEW2871 had differential effects on certain markers such as the stromal cell density; while the former significantly reduced it the latter did not have an effect (Table S1).

To our knowledge, this study is not only the first study to investigate the effect of S1P and its analogs to improve the survival of human OTs, but it is also novel in showing the role of S1P in human transplant angiogenesis in general. As such, while our findings indicate that S1P may improve the success of human OTs by enhancing and accelerating angiogenesis and SC proliferation, they may also lead to further examination of the role of S1P in angiogenesis in other tissues. While our findings justify a clinical trial to prove the clinical benefit of S1P in ovarian transplantation, we need to develop longer acting S1P derivatives or targeted delivery systems to be able to practically administer this agent to patients.

## Materials and Methods

This study was carried out in strict accordance with the recommendations in the Guide for the Care and Use of Laboratory Animals of the National Institutes of Health. The study protocol was approved by the institutional review board of New York Medical College (IRB No. L-9178) and written informed consent was obtained from all participants involved in the study and in case of children, the consent was signed by parents. All surgery was performed under anesthesia, and all efforts were made to minimize suffering. Ovarian tissue fragments were obtained from consenting females patients (n = 5, age range 2–37) undergoing ovarian tissue freezing before chemotherapy. Most of the experiments were performed with fresh tissue to eliminate the possible effect of cryopreservation. Frozen ovarian tissue was only used to study the impact of S1P on follicle survival in frozen-thawed ovarian transplants.

### Human ovarian tissue preparation and transplantation

Human ovarian cortical pieces were prepared as one-mm^3^ pieces and were transplanted into the dorsal muscles of severe-combined-immunodeficient (SCID) mice as previously described [36]. Briefly, ovarian tissue fragments were collected in a modified M199 medium (Gibco, Invitrogen, Carlsbad, USA) while the recipient animals were being anesthetized by Ketamine 75 mg/kg, intraperitoneal (i.p.) (Ketathesia, Dublin, OH, USA) and Xylazine 1 mg/kg i.p. (AnaSed, Shanandoah, Iowa, USA). Analgesia was provided by i.p. injection of buprenorphine 0.1 mg/kg (Buprenex, Reckitt & Colman, Hull, UK) before surgery. A small incision was made in the skin on the dorsal midline of the recipient animal. Using fine watchmakers' forceps, ovarian tissue fragments were randomly picked and xenotransplanted into the dorsal muscles and skin incisions were closed under aseptic conditions.

### Comparison of angiogenic effects of S1P, FTY720 and SEW2871

To compare the angiogenic effects of S1P, SCID mice (n = 8 in each group) xenotransplanted with human ovarian tissue (No. of patients = 3) received either S1P (200 µM)(n = 8 grafts) using Alzet mini-osmotic pumps (Durect, Cupertino, Ca, USA) or FTY720 (2 mg/kg)(n = 12 grafts) (Cayman chemical, MI,USA) or SEW2871 (5 mg/kg)(n = 12) intravenous. Previous work has shown that revascularization of human ovarian xenografts into murine occurs within 3 to 4 days of the transplantation [36]. Therefore, to determine the angiogenic effect of S1P, FTY720 or SEW2871, mice received the drugs for the first 4 days after xenografting. Because S1P has a very short plasma half-life, single dose administration would not be effective. Therefore, a subcutaneous mini-osmotic pump with a flow rate of 8 µl/hour was used to administer S1P (200 µM) continuously for four days via a fine polyethylene catheter tubing (Durect, Cuperino, Ca, USA) directly to the grafts. Mini-osmotic pumps generally need two hours before fully functioning *in vivo*. To provide adequate local concentration of S1P immediately after xenografting, ovarian tissues were placed in the handling medium containing S1P. Immediately before transplantation 200 µM of S1P was injected into the muscular pocket where the xenograft was placed, before closing the skin. Control animals received normal saline via identical osmotic pumps. In the initial experiments we used fresh ovarian tissue, to control for possible effect of cryopreservation on angiogenesis and follicle apoptosis. Grafts were recovered 10 days after xenografting and processed for quantification of angiogenesis, assessment of follicular apoptosis and hypoxia in OTs.

### The impact of S1P on early angiogenesis after xenografting

This experiment was designed based on the data obtained from previous experiment, to characterize the early events occurring during the first days after grafting and administration of S1P. Ovarian tissues were obtained from a 37 year-old consenting female and 12 ovarian tissue pieces were xenotransplanted into the dorsal muscle of SCID mice in each group. Mice received S1P (200 µM) using Alzet mini-osmotic pumps for the first 4 days following xenografting. Control group received normal saline via identical osmotic pumps. Animals were sacrificed daily from days 1–4 and on day 10, and the grafts were recovered. After tissue embedding, tissues were sectioned at 4 µm and were stained for CD31 to determine the vascular density as described later.

### Determining the contribution of recipient site to transplant revascularization using an acellularized extracellular scaffold

We used a human acellularized extracellular matrix scaffold (Alloderm, LifeCell, NJ, USA) to determine whether S1P is enhancing angiogenesis from the murine recipient into human OTs. Alloderm is currently used in practice for the repair of tissue defects, reconstruction, and bridging neovascularization from adjacent tissue edges [54]. 12 pieces of alloderm were xenografted similar to ovarian tissue as described above and S1P was administered for 4 days with osmotic mini-pumps, identical to previous experiments. Alloderm grafts were recovered at day-10. Control animals with similar alloderm xenografts received only normal saline (vehicle). Recovered alloderm grafts were evaluated for quantification of angiogenesis by anti-mouse CD-31 immunohistochemical (IHC) staining (Abcam, MA, USA) and assessment of cellular proliferation by proliferating cell nuclear antigen (PCNA)(Abcam, MA, USA) expression [36], [55].

### Histology, stromal and vascular density assessment, and immunohistochemistry

Histomorphological examination of paraffin-embedded OT grafts was performed after serially sectioning the entire graft at 4 µm. One out of every ten sections was selected for each kind of staining. H&E staining was used to study the tissue architecture, necrosis, and to visualize ovarian stromal cell (SC) for quantification. SC were counted at high-power filed (×400) in a grid area of 0.15 mm^2^ at five randomly selected positions in 25 different sections [36], [56]. IHC staining against PCNA was performed to confirm cell proliferation in the grafted tissues in various experimental groups.

To study neoangiogenesis, epithelial cells of new blood vessels were stained using anti-human CD31 IHC (Ventana, Lille, France). Mature blood vessels were evaluated by means of anti-alpha smooth muscle actin IHC staining (AlphaSMA) (Ventana, Lille, France). To calculate vascular density, CD31 or AlphaSMA staining vessels were counted at high-power filed (×400) in a grid area of 0.15 mm^2^ at five randomly selected positions in 25 different sections [36], [56]. The mean value was used as the final vascular density for each graft.

Apoptotic death was evaluated by anti-active caspase-3 staining (AC3)(R&D systems Minneapolis, USA). Reddish-brown coloring of the cytoplasm/nucleus of the follicles was specified as positive staining. The ratio of apoptotic follicles to total follicular numbers was calculated and compared. Data were compared to baseline ovarian tissue and transplanted vehicle-treated OTs as control.

The level of hypoxia in the grafted tissues were evaluated by the expression of hypoxia inducible factor 1 alpha (HIF1alpha) IHC (Bethyl Laboratories, TX, USA). The ratio of hypoxic area to total surface area of ovarian tissue was compared among different experimental groups.

For negative control, the primary antibody was omitted in each different IHC staining.

### Statistical Analysis

Statistical analysis was performed with the SPSS 17 for Windows package (SPSS Inc., Chicago, IL). Levene's test of homogeneity of variances (p<0.01) and Kolmogorov-Smirnov test of normality (p<0.01) were performed to choose the appropriate statistical test. Continuous data (presented as mean±SD) were analyzed by one-way analysis of variance followed by the LSD post hoc test. The non-parametric data were analyzed with Kruskal Wallis test followed by multiple pair-wise comparisons using Mann-Whitney U-test (α value was adjusted). Fisher's exact test was performed to analyze the relation between two categorical variables. Non-parametric correlation between the percentage of apoptotic follicles and the number of blood vessels was analyzed with the Spearman's test. When the P value was <0.05, the difference was considered statistically significant.

## Supporting Information

Figure S1 **Evaluation of the stromal cell density in ovarian transplants treated with S1P, FTY720, or SEW2871.** Cell counts after H&E staining of ovarian grafts show that S1P results in higher stromal cells density compared to baseline. FTY720 treatment reduces stromal cell density compared to all experimental groups. SEW2871 treatment does not improve stromal cell density and is comparable to grafted vehicle-treated control.
(TIF)

Figure S2 **Evaluation of the impact of S1P on mature blood vessels in ovarian grafts.** (**A-F**) Anti-human αSMA IHC. S1P-treated human ovarian grafts (**D** and **E**) demonstrated lower density of mature blood vessels compared to baseline (**A**) and vehicle-treated controls (**B**). (**F**) CD31 staining of the same vessel as panel **E** (adjacent section) indicates that that S1P can induce neoangiogenesis from pre-existing mature blood vessel. (**G**) Two functioning blood vessels in S1P-treated graft containing red blood cells 10 days post grafting.
(TIF)

Figure S3 **Cell proliferation and migration into alloderm grafts in animals treated with S1P or its analogs.** (**A**–**F**) PCNA staining shows significantly higher proliferation of ovarian stromal cells into alloderm in S1P-treated animals (**D**) compared to vehicle-treated controls (**C**). FTY720 (**E**) and SEW2871 (**F**) do not have an affect compared to vehicle-treated control.
(TIF)

Table S1 Differential impact of S1P and its analogues on ovarian xenografts. ↑: Significantly increased, ↓: Significantly decreased, −: No significant effect.
(DOC)
