# Supplementary figures and images for: Enhancement of Neoangiogenesis and Follicle Survival by Sphingosine-1-Phosphate in Human Ovarian Tissue Xenotransplants
Source: PLoS One. 2011 Apr 29;6(4):e19475. doi: 10.1371/journal.pone.0019475 (PMC3084884; doi:10.1371/journal.pone.0019475)

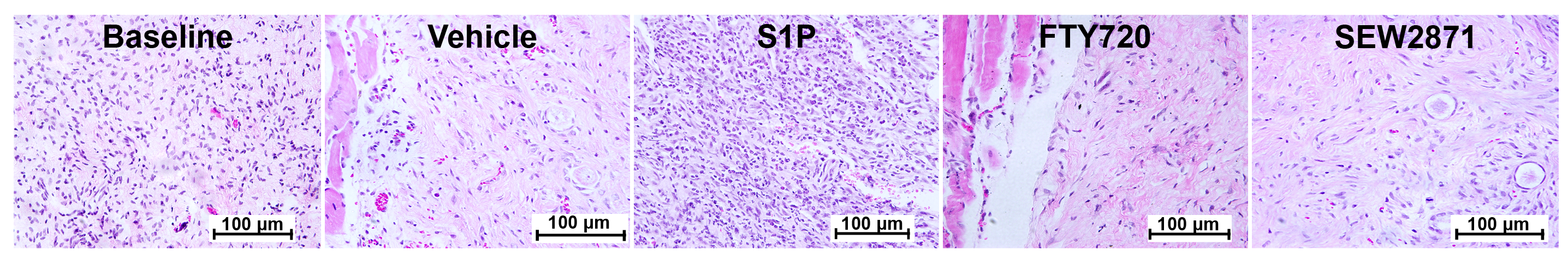

Supplement: Figure S1 — Evaluation of the stromal cell density in ovarian transplants treated with S1P, FTY720, or SEW2871. Cell counts after H&E staining of ovarian grafts show that S1P results in higher stromal cells density compared to baseline. FTY720 treatment reduces stromal cell density compared to all experimental groups. SEW2871 treatment does not improve stromal cell density and is comparable to grafted vehicle-treated control. (TIF) [file pone.0019475.s001.tif]

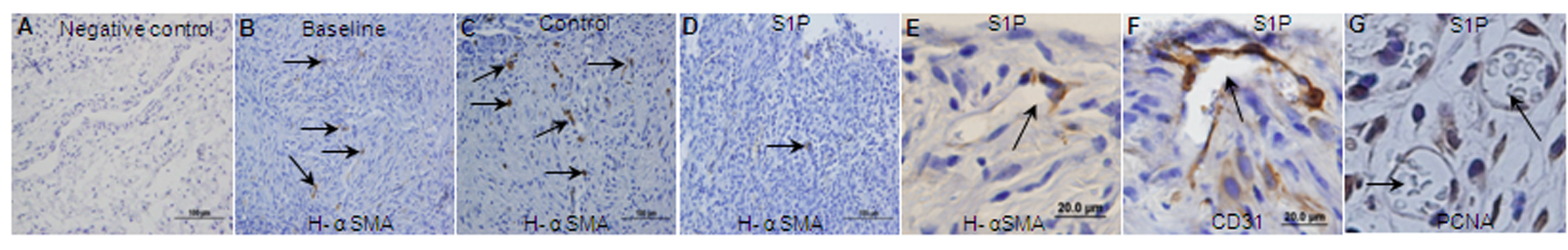

Supplement: Figure S2 — Evaluation of the impact of S1P on mature blood vessels in ovarian grafts. (A-F) Anti-human αSMA IHC. S1P-treated human ovarian grafts (D and E) demonstrated lower density of mature blood vessels compared to baseline (A) and vehicle-treated controls (B). (F) CD31 staining of the same vessel as panel E (adjacent section) indicates that that S1P can induce neoangiogenesis from pre-existing mature blood vessel. (G) Two functioning blood vessels in S1P-treated graft containing red blood cells 10 days post grafting. (TIF) [file pone.0019475.s002.tif]

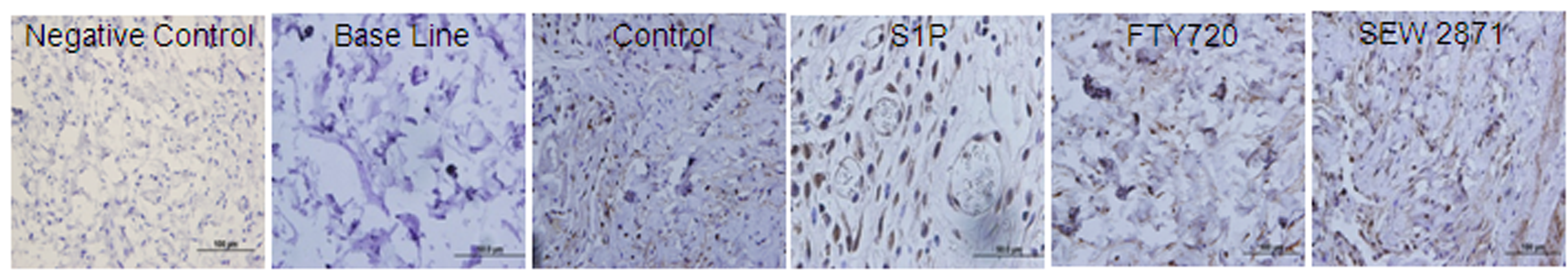

Supplement: Figure S3 — Cell proliferation and migration into alloderm grafts in animals treated with S1P or its analogs. (A–F) PCNA staining shows significantly higher proliferation of ovarian stromal cells into alloderm in S1P-treated animals (D) compared to vehicle-treated controls (C). FTY720 (E) and SEW2871 (F) do not have an affect compared to vehicle-treated control. (TIF) [file pone.0019475.s003.tif]
