# Supplementary material for: Enhancement of Neoangiogenesis and Follicle Survival by Sphingosine-1-Phosphate in Human Ovarian Tissue Xenotransplants
Source: PLoS One. 2011 Apr 29;6(4):e19475. doi: 10.1371/journal.pone.0019475 (PMC3084884; doi:10.1371/journal.pone.0019475)
Supplement: Table S1 — Differential impact of S1P and its analogues on ovarian xenografts. ↑: Significantly increased, ↓: Significantly decreased, −: No significant effect. (DOC) [file pone.0019475.s004.doc]

**Table S1**

|  | S1P | FTY720 | SEW2871 |
| --- | --- | --- | --- |
| Neo-angiogenesis | ↑ | ↓ | ↓ |
| Tissue hypoxia | ↓ | ↑ | ↑ |
| Stromal cell number | ↑ | ↓ | − |
| Follicular apoptosis | ↓ | ↑ | ↑ |
